# Urinary Bladder Hemangioma –A Rare Urinary Bladder Tumor in a Child

**Published:** 2015-01-01

**Authors:** Shubhangi Jibhkate, Vandana Sanklecha, Arvind Valand

**Affiliations:** Department of Pathology, Grant Government Medical College, Mumbai

**Keywords:** Hemangioma, Stem cells, Urinary bladder, Cystectomy

## Abstract

Urinary bladder hemangiomas are rare, accounting for 0.6% of the urinary bladder tumors. Hemangioma is considered arising from embryonic stem cells of an angioblastic lineage. A 3-year old boy presented with hematuria. He had past operative history of excision of extensive lymphatic malformation involving retroperitoneum, pelvis and upper thigh. Computed tomography scan of abdomen and pelvis with contrast revealed a large soft tissue mass arising from the dome of the bladder. Partial cystectomy was done. Histopathology confirmed the mass as cavernous hemangioma of urinary bladder.

## CASE REPORT

A 3-year old male child presented with pain in the lower abdomen of one month duration which was localized and non-radiating along with one episode of gross hematuria. He had past history of extensive lymphatic malformation involving retroperitoneum, pelvis and upper thigh and was operated for the same 18 months back. The lesion was histologically confirmed as lymphangioma. There was no history of trauma, infection or coagulation abnormalities. Physical examination revealed pallor and scar of previous surgery on right upper thigh along with localized tenderness in hypogastric region. Blood work-up was within normal range, however, urinalysis showed 10–20 red blood cells and 2–5 white blood cells per high-power field. Cystoscopy was done from other hospital which revealed a large 6 cm × 5 cm, reddish mass arising from dome of bladder. Computed tomography scan of abdomen and pelvis with contrast also revealed a large 7cm x 6cm x 2cm soft tissue mass arising from the dome of the bladder and hanging in bladder lumen showing homogenous enhancement (Fig.1). There was no evidence of perivesical mesenteric infiltrate.

**Figure:**
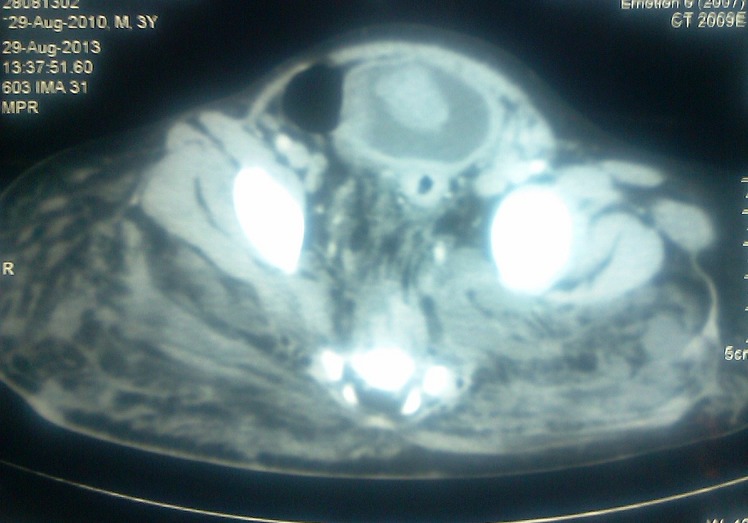
Figure 1: CT scan image shows soft tissue mass arising from dome of the urinary bladder.

Surgery was performed through a lower midline abdominal incision. Urinary bladder was defined by extraperitoneal approach. The tumor was palpated. Partial cystectomy was done and part of bladder containing tumoral vascular tissues with a safe margin was sent for histopathological examination. Macroscopically it was 5cm x 3cm x 2cm partial cystectomy specimen which on cut-section showed a large soft to firm hemorrhagic tumor mass measuring about 2.5cm x 1.6cm x 1cm (Fig.2). Histopathological examination revealed a tumor occupying the submucosa and muscle layer; it was composed of dilated vascular spaces lined by flattened endothelial cells containing red blood cells over-lined by partially ulcerated transitional epithelium. There was no evidence of atypia (Fig.3).Thus final impression of cavernous hemangioma was given on histopathology. Hematuria resolved after surgery and at 1 year follow-up, the patient was doing fine.

**Figure:**
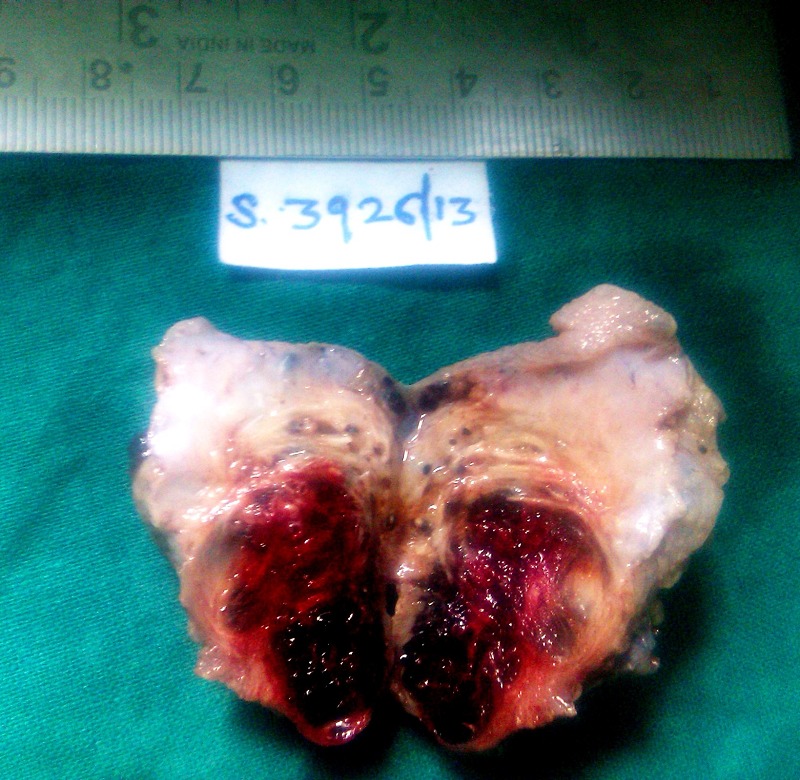
Figure 2: Partial cystectomy specimen which on cut section showed hemorrhagic tumor.

**Figure:**
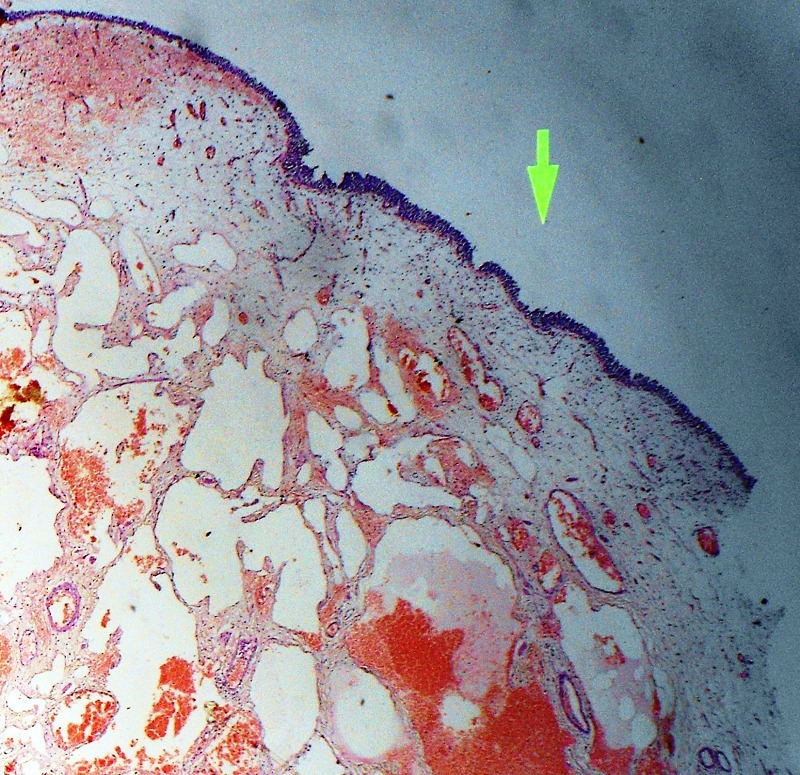
Figure 3: Histopathological examination revealed a hemangioma.

## DISCUSSION

Hemangioma is a common benign tumor occurring in various parts of the body, but it is a very rare primary tumor of the bladder representing only 0.6% of all bladder tumors.[1] It occurs in all age groups but is relatively rare in childhood. It has a slight male predominance.[2] In the review of literature most of hemangioma are solitary (66%), varying from few millimetres to 10cm in diameter with a predilection for the dome, posterior wall, and trigone of the bladder.[2] In most cases, the tumors measure 1 to 2 cm and is sessile. In our case it was large in size.

In most reported cases, it may coexist with a cutaneous hemangioma or be associated with the Klippel-Trenaunay-Weber syndrome where the hemangioma of the urinary bladder are frequent (3-6%).[3] Hemangioma is also associated with Sturge-Weber syndrome or encephalo-trigeminal-angiomatosis, the Rendu-Osler-Weber disease or hemorrhagic telangiectasia syndrome, and systemic angiomatosis.[3] For this reason systemic evaluation in these patients is highly recommended. In our case except for previous scar of lymphangioma excision surgery, systemic evaluation did not reveal any findings.

The predominant clinical symptom of urinary bladder hemangioma is recurrent, isolated hematuria. Other symptoms include suprapubic pain due to vesical irritation and urinary retention.[4] The index case also presented with pain in lower abdomen and gross hematuria.

Management of patients with urinary bladder hemangioma is controversial and should depend on their evolution, size and degree of penetration. Treatment vary from observation, transurethral resection, electrocoagulation, radiation, systemic steroids, injection of sclerosing agent, and partial or complete cystectomy.[5] Asymptomatic hemangiomas do not require treatment. Although bladder hemangioma have a benign course, follow up is important to detect recurrence or residual disease. Severe pain abdomen, hematuria resulting in anemia, and suspicion of some malignant lesion are indications of surgery.

In our case the patient's progressive anemia and large tender vesicle mass led to the decision of surgical tumor removal with a safe margin. Hematuria resolved after surgery and there was no recurrence of bleeding or mass during 1 year follow-up.

## Footnotes

**Source of Support:** Nil

**Conflict of Interest:** None declared

